# Empirically Assessing the Effectiveness of the Pathways Programme: An Online Self-Help Intervention for Male Sexual Aggression at UK Universities

**DOI:** 10.1007/s10508-024-02808-6

**Published:** 2024-02-05

**Authors:** Samuel T. Hales, Caitlyn Rawers, Theresa A. Gannon

**Affiliations:** 1 School of Psychology, University of Kent, Canterbury, CT2 7NP UK; 2 School of Psychology, Ulster University, Coleraine, UK

**Keywords:** Sexual aggression, Harm prevention, Higher education, Self-help interventions, Sexual violence

## Abstract

Though contemporary evidence suggests that upwards of one-in-nine UK male university students engage in sexually violent behaviors (Hales and Gannon in Sexual Abuse 34:744–770, 2022), few evidence-based primary prevention strategies have been developed to prevent their perpetration. To help contribute to this evidence gap, this study evaluated the short and longer-term effectiveness of a novel psychoeducation-based online self-help intervention for university male sexual aggression called The Pathways Programme. Designed around current empirical understanding of university-based sexual harm in the UK, the program contains six modules that correspond with known risk factors for perpetration. Data were collected as part of a randomized control trial from 254 self-identified heterosexual male students enrolled at a UK university who reported a proclivity towards sexual aggression. Results showed that participants who took part in the program displayed moderate reductions in their self-perceived likelihood of sexual aggression (our primary treatment target) across testing points, as well as reductions in their self-reported levels of hostility towards women, rape myth acceptance, and problematic sexual fantasies (our secondary treatment targets). Control participants also displayed reductions in some domains over time, albeit to a lesser degree. Additional analyses probed the factors associated with participant drop-out, clinical and reliable change, and user feedback. Overall, our study provides preliminary evidence for the efficacy of The Pathways Programme at reducing UK university males’ risk of sexual aggression; however, we caution readers that more robust evaluation is necessary to support intervention rollout. We discuss our findings alongside the limitations of our study and provide suggestions for future research.

**Supplementary Information:**

The online version contains supplementary material available at 10.1007/s10508-024-02808-6.

## Introduction

University-based sexual aggression is a global public health issue associated with negative physical, psychological, and academic outcomes (Jones et al., 2020; Molstad et al., 2021). Conceptualized as a form of gender-based violence (GBV), university-based sexually aggressive offences span a spectrum of non-consensual sexual activities that predominantly target female students (Fedina et al., 2018). One-in-five US female students report being a victim of university-based sexual aggression (Muehlenhard et al., 2017), with a quarter disclosing victimization in the past two-months (Jouriles et al., 2022). Though these worrying statistics have been replicated in other countries (Hernández-Romero et al., 2019), high rates of under-reporting and discrepancies across sexual aggression measures mean that they are likely conservative estimates (see Bouffard & Goodson, 2017; Fedina et al., 2018).

While there have been relatively few assessments of sexual aggression perpetration on university campuses, climate survey data have shown that male students commit the majority of offences (Anderson et al., 2021; Jones et al., 2020). This finding has been validated across several large-scale studies with male students, where approximately one-in-four report a history of sexual perpetration (Anderson et al., 2021) and notably more report a proclivity towards harmful sexual activity (e.g., Palmer et al., 2021; Wong et al., 2020; Zounlome & Wong, 2019)—a strong indicator of future campus-based sexual offending (e.g., Malamuth et al., 1995; Zounlome & Wong, 2019). Worryingly, despite recent federal guidelines to make campuses safer, self-reported perpetration rates among male US student samples have increased over the past three decades (Koss et al., 2022).

In spite of modern policy advances and investment in sexual harm prevention (see Donaldson et al., 2018), recent climate survey evidence has shown that 70% of female students and recent graduates in the UK experience sexual violence at university, with 8% reporting rape victimization during their studies (Revolt Sexual Assault [RSA], 2018). Similar rates have been reported in follow-up surveys (e.g., Brook, 2019; Camp et al., 2018), including a recent investigation by Tutchell and Edmonds (2020) which suggested that over 50,000 UK university students experience sexual assault annually. Mirroring international findings, male students perpetrate the majority of offences on UK campuses (Jones et al., 2020). However, a lack of robust reporting standards in this area (see Giroux et al., 2020), as well as the aforenoted known issue of under-reporting sexual offending behaviors, mean that evaluations of sexual violence prevalence at UK universities are likely to underestimate the scope of the issue. This point is underlined by Bull et al. (2022) who, in reflecting on the political and methodological challenges they faced when conducting climate surveys at their academic institutions, noted that a lack of precedence for measuring university-based sexual aggression in the UK has, to date, impeded the collection of meaningful and objective offending data. Indeed, Bull et al. highlighted that there is no sector standard in terms of reporting sexual violence prevalence rates in the UK, which may account for the notable discrepancies in self-reported (victimisation and perpetration) prevalence estimates among UK university students.

### Key Risk Factors for University-Based Sexual Aggression: An International Perspective

International research has identified several risk factors associated with university males’ harmful sexual behaviors (see O’Connor et al., 2021; Spencer et al., 2023; Teten Tharp et al., 2013; Thompson et al., 2013, 2015). These span the social ecology and often work synergistically to encourage sexual perpetration (Bonar et al., 2022; McMahon et al., 2019). While risk factors vary between perpetrators, contemporary work has validated several key factors as strong indicators of male students’ offending behaviors (for a review, see Hales, 2022; O’Connor et al., 2021; Spencer et al., 2023). Of these factors, researchers have established that gender-based cognitions, sex-related cognitions, and lack of knowledge about sexual consent are key risk factors for university-based sexual aggression (for a review, see Hales, 2022).

Gender-based cognitions typically refer to male students’ prejudicial and sexist attitudes towards female students. Most work into gender-based cognitions derives from feminist theory and asserts that men’s sexual aggression stems from patriarchal social structures designed to control women (see Murnen et al., 2002). In support of this, studies have reported that many US male students with histories of sexual aggression possess hostile attitudes towards women and endorse common rape myths (O’Connor et al., 2021; Yapp & Quayle, 2018)—known “hostile masculine” traits that have been shown to work in concert with other risk factors to encourage men’s perpetration (see Ray & Parkhill, 2021).

Empirical support has also been provided for sex-related cognitions as predictors of students’ harmful sexual behaviors. For example, studies have shown that male students in the US who possess problematic sexual fantasies are more likely to engage in illegal sexual activities compared to those without such fantasies (e.g., Gold & Clegg, 1990; Malamuth et al., 1995; Williams et al., 2009).^1^

Misunderstanding of sexual consent also constitutes a key risk factor for university-based sexual aggression in the US. In support of this, research has shown that US students who are unable to identify appropriate indicators of valid sexual consent, including those who endorse non-verbal consent strategies, are more likely to engage in non-consensual sexual activities (e.g., Salazar et al., 2018; Walsh et al., 2021; Zinzow & Thompson, 2019).

### Understanding Sexual Perpetration by UK Male Students

Compared to the US, academic understanding of university-based sexual aggression in the UK is underdeveloped (see Jones et al., 2020). An empirical review by Hales and Gannon (2022) found that one-in-nine UK university males report recent sexual aggression perpetration. The authors also discovered that students who had not perpetrated an offence were psychologically different from self-reported perpetrators, whose offending histories could be predicted by their levels of hostility towards women, rape myth acceptance (RMA), and problematic sexual fantasies.

Hales and Gannon’s (2022) findings align well with other studies conducted with UK male students, which have also signalled individual-level risk factors for sexual aggression. For example, “lad culture”—defined as “a group or ‘pack’ mentality residing in activities such as sport and heavy alcohol consumption, and ‘banter’ which [is] often sexist, misogynist and homophobic” (Phipps & Young, 2013, p. 28)—has been extensively reviewed as an explanatory factor for GBV at UK universities (e.g., Diaz-Fernandez & Evans, 2020; Jackson & Sundaram, 2020; Jeffries, 2020). In their ground-breaking *That’s What She Said* report, Phipps and Young (2013) noted that “laddism” can be conceptualized as one of several harmful forms of masculinity that shape the identities and attitudes of male students. Phipps and Young note that lad culture is perceived by many male students as the “template” of UK masculinity, with several men engaging in deviant “laddish” activities in order to fit in with their peers. This is a worrying notion, particularly given that a significant number of university males in the UK report that laddism is a ubiquitous part of their university lives (Jeffries, 2020).

Beyond lad culture, research has highlighted that many UK male students also self-report high levels of hostile sexism (Davies et al., 2012), physical (non-sexual) aggression (Bhogal & Corbett, 2016), and RMA (e.g., Bhogal & Corbett, 2016; Camp et al., 2018)—other hostile masculine indicators of sexual aggression. In a recent study, Samji and Vasquez (2020) reported strong links between UK male students’ hostility towards women, RMA, and sexual objectification, providing further support for the notion that hostile masculine traits are likely to constitute a potent set of risk factors for university students beyond the US.

Beyond individual-level cognitions, recent academic research has explored how university students across the country perceive and comprehend sexual consent. For example, across several vignette studies, Hills et al. (2020) found that many UK male students determine whether sexual activity meets the threshold for sexual aggression based on a victim’s apparent desire for sex and their outward expressions of sexual pleasure. These findings reflect those reported by Wignall et al. (2020), who found that many university students across the country interpret an absence of a “no” as a valid indicator of consent in sexual situations.

### University Sexual Harm Prevention in the UK

Unlike the US, where universities are mandated to deliver prevention programs for sexual aggression, university-based sexual harm prevention work in the UK is in its infancy. A recent report published by Universities UK (UUK, 2016)—an advocacy organization for higher education providers in the UK—encouraged universities to prioritize tackling GBV and develop more evidence-based prevention interventions to help reduce high rates of sexual perpetration by students. This call-to-action was supplemented by catalyst funding awarded to 63 universities by the Office for Students—the national independent regulator of higher education—to formulate more effective strategies to reduce sexual aggression on campuses nationwide (HEFCE, 2017).

In 2017, UUK showcased examples of sexual harm prevention initiatives being adopted by higher education institutions across the UK (UUK, 2017). This case study report highlighted that several universities were taking proactive steps to protect students from committing sexual harm by implementing preventative campus-wide measures. However, the report also underscored a disparity in approaches used (see Baird et al., 2019), as well as a lack of evidence-base for effective program development.

It is worth noting that some effective prevention programs do exist in the UK. For example, bystander programs—a popular form of community-based prevention intervention in the US (see Kettrey & Marx, 2019)—have been implemented across several UK campuses (see Chantler et al., 2019; UUK, 2017, 2018). Noteworthy examples of bystander programs are available (e.g., *The Intervention Initiative*; Fenton et al., 2014) that bring about positive short-term shifts in students’ behaviors and attitudes (e.g., Fenton & Mott, 2018; Roberts & Marsh, 2020). However, these programs are often modelled on US data that may not generalize to UK students due to obvious differences in history, culture, and geography between both countries. Although bystander programs have been implemented in UK universities, data on the long-term impact of these interventions on individual behavior change is limited (see Baird et al., 2019). Likewise, the programs place the onus on the broader university community, rather than perpetrators, to reduce GBV. As Camp et al. (2018) note, this means that current bystander interventions likely do not target those students who are most at risk of offending.

Internationally, other university-based sexual harm prevention programs have been developed that have earned preliminary support. For example, in Vietnam, a web-based intervention covering topics including consent, RMA, and hostile masculinity was delivered to male university students and led to a decrease in sexual violence perpetration and an increase in bystander intervention (Yount et al., 2023). Likewise, participants who received a German intervention designed to address risk and vulnerability factors related to sexual aggression reported medium to long-term reductions in risky sexual scripts and subsequent behavior as well as increased sexual self-esteem and assertiveness compared to a “no-intervention” control group (Schuster et al., 2023). At several US universities, students who perpetrate sexual harm are referred to the Science-based Treatment, Accountability, and Risk Reduction for Sexual Assault (STARRSA) program for a cognitive behavioral therapy or psychoeducation-based intervention to address their sexual misconduct (see Lamade et al., 2018). To date, however, no evaluation data have been published on the effectiveness of STARRSA for addressing sexual harm perpetration.

### Rationale for Our Study

Many university-based sexual harm prevention interventions in the UK lack theoretical support and UK-based empirical evidence, despite emerging research on the risk factors associated with students’ harmful sexual behaviors. Few have also been longitudinally evaluated, meaning that their efficacy at producing long-term behavioral or attitudinal shifts is undetermined. Of the limited empirically-informed programs that do exist, most are guided by US research. While these are likely to be broadly relatable to the UK context, disparities in university history, culture, and geography between both countries (see Jones et al., 2020), mean that prevention programs informed only by empirical work with students in the US may neglect important factors unique to the UK context (see Labhardt et al., 2017).

Our study contributes to the current gap in UK university-based sexual harm prevention research by evaluating the feasibility and efficacy of The Pathways Programme—a novel online self-help intervention for university male sexual aggression designed using psychological theory and emerging empirical evidence relevant to university-based sexual aggression perpetration by UK male students. In presenting and evaluating The Pathways Programme, we not only contribute data to the growing knowledge base on university-based sexual aggression in the UK, but we also respond to UUK’s call for the development of more UK-centric evidence-based prevention programs to tackle campus sexual perpetration.

### Study Aims and Hypotheses

The primary aim of our study was to evaluate the short and longer-term effectiveness of The Pathways Programme at reducing participants’ self-reported proclivity to engage in sexually aggressive behavior. Proclivity was considered our primary outcome measure as it is a more reliable indicator of future offending behaviors than past perpetration, which can be unreliably assessed via self-report measures (see Anderson et al., 2021). Likewise, proclivity is more proximal to sexual perpetration than our secondary outcome measures, which signal broader attitudinal and behavioral risk factors for university-based sexual aggression. While self-reported proclivity is not a perfect indicator of future sexual perpetration, several studies have established a strong link between both factors (e.g., Malamuth et al., 1995; Palmer et al., 2021; Zounlome & Wong, 2019).

Further to our primary aim, we also sought to assess the degree to which The Pathways Programme could engender positive treatment shifts across three psychological domains highlighted by Hales and Gannon (2022) as key risk factors for UK male university sexual perpetration. These included students’ self-reported hostility towards women, RMA, and problematic sexual fantasies.

Based on the well-reported issue of high student drop-out rates across longitudinal sexual aggression studies (e.g., Salazar et al., 2014; Wong et al., 2020), we further explored predictors of participant retention in our intervention using Ajzen’s (1991) theory of planned behavior (TPB), which hypothesizes that behavior (in this case, participation in our intervention) is best predicted by an individual’s behavioral intentions and normative or control-based beliefs.

## Method

Our study adopted a randomised control trial (RCT) design to assess the short-term (i.e., pre/post) and longer-term (i.e., 3-month) effectiveness of The Pathways Programme. Our research design allowed us to evaluate the feasibility and efficacy of the program across a cohort of UK male university students in both a timely and cost-effective manner. Assessing participants’ scores 3-months after they took part in the program allowed us to examine any possible rebound effect—an established phenomenon associated with sexual harm prevention interventions in which participants display large attitudinal shifts immediately post-intervention but not over longer periods (see DeGue et al., 2014).

Data collection for this study took place in waves between April and November 2021. The evaluation comprised four standalone studies that ran sequentially: a pre-test survey, the intervention (completed by half of the sample), a post-test survey, and a 3-month follow-up survey. Our hypotheses, method, and data analysis plan were pre-registered at https://osf.io/b79n3/, where readers can access copies of our intervention, study materials, and raw data.^2^

### Participants

**Fig. 1:**
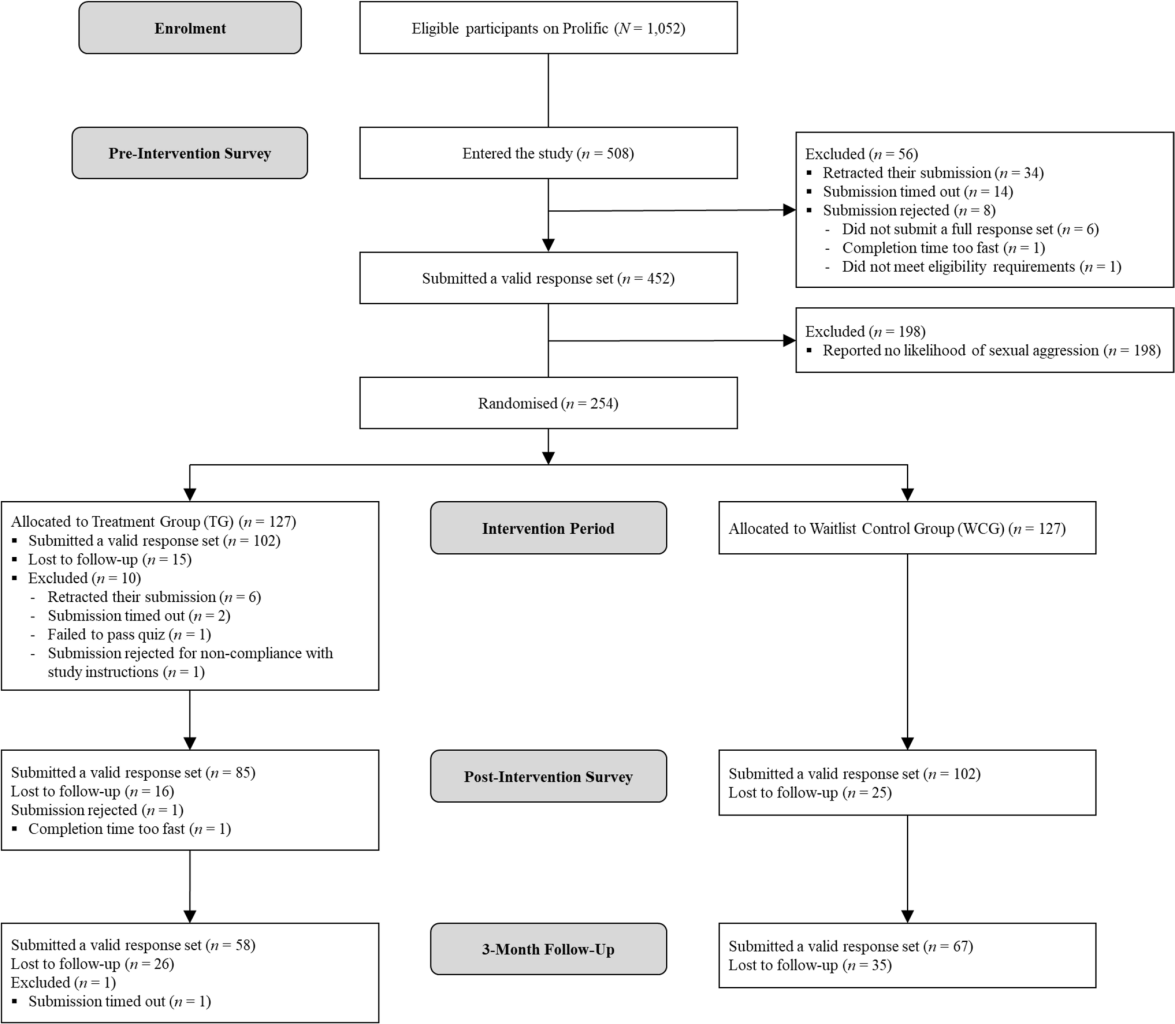
A CONSORT diagram detailing the flow of participants through the study

Participants were recruited through Prolific (see Palan & Schitter, 2018)—a popular crowdsourcing site that has received positive academic evaluation by sexual harm researchers (Ó Ciardha et al., 2021). Pre-screening filters were set for age (18 +), sex (male only), sexual orientation (heterosexual), student status (students only), and current level of study (undergraduate, postgraduate, and doctorate degree level). We specified that participants should have a Prolific account registered in the UK. Therefore, our study was only visible to male UK heterosexual university students and identified an eligible target population of 1,052 students. A priori power analyses showed that, based on an α error level of 0.05 and 80% power, at least 80 participants were required overall to detect a medium effect size in our planned mixed model analyses. Given the established high rates of attrition in sexual harm prevention studies and general sexual offence treatment (e.g., Olver & Wong, 2011; Salazar et al., 2014; Wong et al., 2020), as well as the relatively low number of UK male students who report sexual aggression (see Hales & Gannon, 2022), we recruited more participants than suggested by our power analysis. In total, 452 participants took part in our pre-test survey, entitled “Promoting Healthy Sexual behaviors on Campus: A Longitudinal Assessment of a Novel Self-Help Intervention.” Of these, 198 reported no likelihood of sexual aggression (as identified by their emphatic rejection of sexual aggression-related items on our primary outcome measure); therefore, our final sample comprised 254 participants (see Fig. 1 for a CONSORT diagram).

There were descriptive similarities between the demographic characteristics of our participants and the broader UK male student body (see HESA, 2022). Participants’ ages ranged from 18 to 78 years (*M* = 25.77, *SD* = 7.93; see Supplemental Table 1). The majority identified as “White British” (*n* = 138; 54.3%) and reported their current level of university study as “undergraduate or equivalent” (*n* = 162; 63.8%). In terms of relationship status, most participants reported that they were “single or self-partnered” (*n* = 133; 52.4%), though a noteworthy proportion did self-report having a “partner” or “wife” (*n* = 120; 47.2%). Overall, participants from 91 different UK universities were represented in this study.

### Measures

Across surveys, participants completed four validated self-report measures relevant to the primary and secondary study outcomes. Two additional measures were administered to participants who took part in the intervention to ascertain their research motivations and perceptions of the intervention. We relied on validated short-form measures where possible to mitigate against participant fatigue. Select items were rephrased to increase their relevance for UK students (e.g., “college” was changed to “university”).

Across measures, Cronbach’s alpha (*α*) was calculated as a metric of internal consistency; scores were interpreted using George and Mallery’s (2016) criteria. Test–retest reliability was computed for participants who did not complete the intervention (and thus were not expected to display any treatment shifts) using intraclass correlation coefficients (ICC), which were based on a mean-rating (*k* = 3), absolute-agreement, two-way mixed-effects model. ICC scores were interpreted using Koo and Li’s (2016) guidelines. Across studies, all measures displayed “excellent” test–retest reliability.

#### Primary Outcome Measure

**Self-Perceived Likelihood Scale** (SPLS; Zounlome & Wong, 2019). We used a modified version of the SPLS to assess participants’ self-perceived likelihood (i.e., proclivity) of sexual aggression. The SPLS comprised six items each describing a specific sexually aggressive act (e.g., “Raping an adult female”).^3^ These items were presented alongside ten non-sexual filler items (e.g., “Driving 130 MPH on the motorway”). Using a 5-point Likert scale anchored by 1 (*Very unlikely*) and 5 (*Very likely*), participants rated how likely they would be to engage in each behavior if they could be assured that there would be no consequences. Responses were averaged across items for a single composite score ranging from 1 to 5. Higher scores reflect an increased likelihood of sexual perpetration.

The developers of the SPLS reported that the scale demonstrates “acceptable” to “excellent” internal consistency with undergraduate male students in the US (Wong et al., 2020; Zounlome & Wong, 2019). Likewise, it converges with measures of past sexual aggression and known indicators of UK male students’ sexual perpetration (Zounlome & Wong, 2019). In our study, internal consistency scores for the SPLS were “good” at all three testing points (*α* = 0.82–0.87). The ICC score was 0.92, 95% CI [0.89 to 0.94].

#### Secondary Outcome Measures

**Hostility Towards Women Scale** (HTW; Lonsway & Fitzgerald, 1995). Participants’ endorsement of hostile and sexist attitudes towards women were assessed using the 10-item HTW. Responses were made on a 7-point Likert scale anchored by 1 (*Strongly disagree*) and 7 (*Strongly agree*). Sum scores were generated for a total score that could range from 10 to 70. Higher scores reflect more hostile perceptions of women. In our study, internal consistency scores for the HTW were “good” to “excellent” at all three testing points (α = 0.88–0.91). The ICC score was 0.95, 95% CI [0.94 to 0.97].

**Illinois Rape Myth Acceptance Scale—Revised** (IRMA-R; McMahon & Farmer, 2011). The 19-item IRMA-R was used to assess participants’ endorsement of subtle myths pertaining to rape and sexual assault. Responses were made using a 5-point Likert scale from 1 (*Strongly disagree*) to 5 (*Strongly agree*) and were summed for a total score that could range from 19 to 95. Higher scores reflect greater acceptance of rape myths. In our study, internal consistency scores for the IRMA-R were “excellent” at all three testing points (*α* = 0.91–0.94). The ICC score was 0.93, 95% CI [0.91 to 0.95].

**Sexual Fantasy Scale Revised—Short Version** (SFQ-R-SV; Bartels & Harper, 2018). To examine problematic sexual fantasies, participants responded to 27 items from the Masochistic, Sadistic, Impersonal, and Pre/Tactile Courtship Disorder subscales of the SFQ-R-SV. Both the Romantic and Bodily Functions subscales were not included as the former does not refer to problematic sexual fantasies and the latter is endorsed infrequently in community samples (Bartels & Harper, 2018). Responses were made on a 5-point Likert scale from 0 (*Have never fantasized about*) to 4 (*Have fantasized about very frequently*). Total scores could range from 0 to 108, with higher scores indicating greater endorsement of the described fantasies. In our study, internal consistency scores for the SFQ-R-SV were “good” to “excellent” at all three testing points (*α* = 0.88–0.90). The ICC score was 0.93, 95% CI [0.91 to 0.95].

#### Participant Engagement

**Theory of Planned Behavior Questionnaire** (TPBQ; Wojtowicz et al., 2013). To examine the psychological factors associated with intervention completion, we administered to participants the TPBQ (Wojtowicz et al., 2013). The scale was modified to refer to The Pathways Programme specifically and administered only to participants who took part in this Programme (see Supplemental Appendix A). The measure comprised ten items (four reverse-coded) apportioned across four subscales that quantitively assessed each domain of the TPB (i.e., attitudes, intentions, perceived behavioral control, and subjective normative beliefs). Participants responded to items on a 7-point Likert-type scale and composite scores were generated for each subscale. As such, subscale scores could range from 1 to 7. To mask its aims, the TPBQ was presented as a “user engagement survey.” Wojtowicz et al. (2013) did not report internal consistency for the TPBQ in their study. In our study, internal consistency was “good” overall (*α* = 0.81) and “questionable” to “acceptable” for each subscale (*α* = 0.63–0.78).

#### User Feedback

**User Feedback Measure** (Thompson et al., 2021). Participants’ perceptions of The Pathways Programme were assessed using a feedback measure adapted from Thompson et al. (2021). The measure comprised 15 items (one reverse-coded) that participants responded to on a 7-point Likert-type scale anchored by 1 (*Not at all true*) and 7 (*Very true*). A composite score was calculated that could range from 1 to 7, with higher scores reflecting more positive user feedback. A follow-up item asked participants for qualitative feedback. In our study, internal consistency for the user feedback measure was “excellent” (*α* = 0.94).

### The Intervention

The Pathways Programme is a psychological self-help intervention designed to reduce UK male university students’ proclivity towards engaging in harmful sexual behaviors. The intervention is predominantly psychoeducation-based, though includes cognitive behavioral activities designed to stimulate positive behavior change. The intervention is modular in format and self-administered by participants online via the secure survey-hosting site Qualtrics. Module content reflects current academic understanding of UK male perpetrators’ university-based sexual violence (e.g., Hales & Gannon, 2022), as well as effective sexual harm prevention strategies (e.g., Bonar et al., 2022; DeGue et al., 2014). The Pathways Programme was set up such that participants were able to pause their activity and return at a later stage without losing their progress.

The Pathways Programme comprises six core modules and one optional module that are worked through sequentially (for an overview, see Supplemental Appendix B). Module content overlaps with areas of concerns identified recently by Humphreys and Towl (2020) in their “good practice guide” for university-based sexual harm prevention. The first three modules reflect the key treatment target of the intervention (i.e., sexual harm proclivity) while the last three modules map onto known psychological risk factors for sexual aggression among UK university males (see Hales & Gannon, 2022). An optional module on mindfulness meditation is offered at the end of the intervention to help alleviate psychological distress.

In terms of design, modules are mostly text-based and follow a workbook format that includes psychoeducation, interactive quizzes, links to further resources, and applied activities. Quizzes assess participants’ understanding of module content, while activities encourage participants to apply their learning to real-world scenarios. Quizzes are multiple-choice and provide participants with instant feedback. A “spotlight” section is included during each module to help reaffirm key lessons. Modules were 10 to 20-min in length—though can be much longer if participants engage fully with the further resources—for an estimated overall completion time of 80-min. Metadata collected via Prolific showed that the median completion time for the intervention was 70-min, indicating good participant engagement with the intervention.

### Procedure

#### Pre-Test Survey

Eligible participants accessed our pre-test survey, hosted on Qualtrics, via their Prolific dashboard. Participants initially completed a screening measure to corroborate their responses to Prolific’s pre-screening filters, before responding to a demographic survey and then our primary and secondary outcome measures. With the exception of the SPLS (which was presented last), measures were presented randomly. To ensure a complete response set at each wave, the survey was set up so that participants had to respond to all items.

At several points, participants’ SPLS responses were reviewed by the authors. As they would not benefit from the intervention, participants who rejected all six SPLS items (i.e., they did not self-report a harmful sexual proclivity) were excluded from the study. Data collection then continued, following this iterative process, until an appropriate sample size was reached. Regular random spot-checks of completed surveys were performed to ensure that there were no obvious engagement violations.

#### Intervention Period

As part of our RCT design, participants were randomly, but equally, allocated to either a treatment group (TG) or a waitlist control group (WCG) using free online software (https://www.random.org/lists). TG participants received immediate access via a private link to The Pathways Programme, which they had four weeks to complete. TG participants were told that their engagement in the intervention would be monitored and that they could be excluded from the research if they were found to be non-compliant. Conversely, WCG participants were thanked for their participation in our pre-test survey and told that they had been placed on a waitlist for the “heavily subscribed” intervention. The intervention was presented as “a novel intervention that is being trialled to provide education to help promote healthy sexual behaviors on campus.”

#### Post-Test Survey

After four weeks, participants in both groups re-completed each of our primary and secondary outcome measures. WCG participants were told that the purpose of this survey was to re-assess their eligibility to take part in The Pathways Programme, while TG participants were told that the survey was designed to assess program effectiveness.

#### Follow-Up Survey

After three months, TG and WCG participants were contacted one final time and asked to re-complete the post-test survey. Participants were told that the purpose of this survey was to assess any shifts in their attitudes over time. At this point, the WCG had not undertaken any participation in The Pathways program.

### Analysis Plan

Analyses were conducted using SPSS v.28 for Windows (IBM Corp., 2021). Consistent with our pre-registration, intervention quiz scores were reviewed prior to analysis to ensure that each participant surpassed the 70% threshold for acceptable user engagement. One participant who scored less than 70% across quizzes was removed from our dataset.^4^

Intervention effectiveness was examined in three ways. First, to assess the ability of The Pathways program to influence participants’ scores across each outcome measure over time, we conducted a series of two-way mixed models that accounted for the repeated measures design of our research. Group allocation was defined as the between-subjects factor and testing point was defined as the within-subjects factor. Partial eta squared was used as a measure of effect size and scores were interpreted using Cohen’s (1988) guidelines. Significant interaction effects were assessed via a series of Wilcoxon signed-rank tests.

The second criterion for evaluating efficacy was clinical significance (see Jacobson et al., 1984), which allowed us to examine individual-level changes in self-reported proclivity towards harmful sexual activity among participants in both the TG and WCG. A participant was classed as exhibiting clinically significant change (CSC) if their composite SPLS score shifted from > 1 (reflecting a non-zero endorsement of at least one SPLS item) at pre-test to 1 (reflecting an emphatic rejection of all SPLS items) at either post-test or follow-up.

Finally, a variant of Jacobson and Truax’s (1991) reliable change indices (RCI) were calculated to assess whether the effects of The Pathways program were reliable at each testing point. Reliable changes in sum scores for each of the outcome measures from pre-test to post-test/follow-up were evaluated separately for TG and WCG participants using the following formula:

$$RCI= \frac{{X}_{2}-{X}_{1}}{{SE}_{diff}} \; {\text{where}} \; {SE}_{diff}= \sqrt{2({SE)}^{2}} \; {\text{and}} \; SE= {SD}_{pre}\times \sqrt{1-{ \alpha }_{pre}}$$

where *X*_2_ = post-test/follow-up score; *X*_1_ = pre-test score; SE_diff_ = standard error of differences; SE = standard error; SD_pre_ = standard deviation at pre-test; and *α*_pre_ = Cronbach’s alpha of pre-test. Participants with an RCI score ± 1.96 displayed a score change that was unlikely to occur solely due to the unreliability of the measure; thus, their change is considered ‘reliable’ (Evans et al., 1998). If participants had a RCI score of − 1.96, they had a reliable reduction in their sum scores from pre-test to post-test/follow-up. Conversely, an RCI score of + 1.96 reflected a reliable increase in sum scores at post-test/follow-up.

For the primary outcome measure, participants were classified into one of five categories of reliable change based on clinical significance and their RCI score: “recovered” (a reliable and clinically significant reduction in SPLS scores), “reliable improvement” (a reliable, but not clinically significant, reduction in SPLS scores), “unreliable recovery” (a clinically significant, but not reliable, reduction in SPLS scores), “unchanged” (no reliable or clinically significant change in SPLS scores), or “deteriorated” (a reliable increase in SPLS scores).^5^ For the secondary outcome measures, participants were classified into one of three groups based on their RCI scores: “improved” (a reliable reduction in sum scores), “unchanged” (no reliable change in sum scores), and “deteriorated” (a reliable increase in sum scores). For primary and secondary outcome measures, a classification of “unchanged” does not reflect absolutely no change in sum scores between testing points; rather, it shows that the change was not significant according to RCI criteria (i.e., it was less than ± 1.96). Chi-square analyses compared the proportion of participants from the TG and WCG in each category at post-test and follow-up.

We followed an intention-to-treat (ITT) approach when analysing data. While we inspected for and responded to unusual data points across statistical tests, we did not remove outliers a priori. As such, our final sample comprised all 254 participants from our pre-test survey. Concurrent with ITT principles, missing data were imputed using the expectation maximisation approach—an iterative procedure in which missing data are estimated by calculating the log-likelihood function of other available parameters.

Across outcome measures, data were found to be missing completely at random using Little’s (1988) omnibus test, *χ*^2^ (12) = 16.71, *p* = 0.161.

## Results

### Adherence and Attrition

Attrition rates were recorded both overall and separately for the TG and WCG across testing points (see Fig. 1). Attrition was defined as the number of participants who withdrew from the study, were lost to follow-up, or were excluded since pre-test. Results showed that overall attrition was 26.4% (*n* = 67) at post-test and 50.8% (*n* = 129) at follow-up. Thus, roughly half (49.2%; *n* = 125) of our pre-test sample completed our study in full. Drop-out rates at follow-up did not significantly differ between groups, *p* = 0.26.

We examined possible differences between completers and non-completers in baseline scores across demographic and outcome variables. Results revealed that non-completers were significantly younger (*U* = 6384.00, *p* = 0.004, *z* = − 2.88), undertaking a lower level of current university study (*p* = 0.03, Fisher’s exact test), and had higher baseline HTW scores (*U* = 9397.00, *p* = 0.02, *z* = 2.28) and IRMA-R scores (*U* = 9402.00, *p* = 0.02, *z* = 2.29) compared to completers.

Consistent with CONSORT guidelines for RCTs (Moher et al., 2010), we did not include participants’ baseline HTW or IRMA-R scores as covariates in our mixed model tests. However, we did control for participants’ age and current level of university study, given their established link to students’ sexual aggression (e.g., Abbey et al., 2001; Porta et al., 2017; Thompson et al., 2013).

### Baseline Equivalence Between Groups

Despite randomisation, analyses revealed group differences in pre-test scores. The TG had significantly higher SPLS scores (*U* = 6835.00, *p* = 0.03, *z* = − 2.13), IRMA-R scores (*U* = 6877.50, *p* = 0.04, *z* = − 2.03), and SFQ-R-SV scores (*U* = 6619.00, *p* = 0.01, *z* = − 2.47) compared to the WCG. Again, following CONSORT RCT guidelines (Moher et al., 2010), we did not control for these baseline imbalances in our mixed model tests.

### Primary Intervention Outcomes

Table 1 contains descriptive statistics for the SPLS (both individual items and the overall scale) between TG and WCG participants across each testing point. Results of our Wilcoxon signed-rank tests are shown in Table 2. Across significance tests, *r* was used as an effect size.

**Table 1:** Mean composite scores on the Self-Perceived Likelihood Scale (SD)

Item	Treatment group (*n* = 127)			Waitlist control group (*n* = 127)		
	Pre-test (*n* = 127)	Post-test (*n* = 85)	Follow-up (*n* = 58)	Pre-test (*n* = 127)	Post-test (*n* = 102)	Follow-up (*n* = 67)
Raping an adult female	1.28 (0.80)	1.16 (0.46)	1.12 (0.33)	1.09 (0.43)	1.15 (0.50)	1.19 (0.68)
Forcing an adult female to do something sexual that they don’t want to do	1.49 (0.93)	1.34 (0.73)	1.21 (0.45)	1.27 (0.61)	1.26 (0.63)	1.34 (0.83)
Having sex with an adult female who is incapacitated	1.40 (0.88)	1.21 (0.54)	1.19 (0.51)	1.25 (0.70)	1.25 (0.61)	1.31 (0.86)
Having sex with an adult female you just met who looks like she has been flirting with you but hasn’t verbally agreed to it	2.44 (1.10)	2.06 (1.16)	1.81 (1.02)	2.46 (1.13)	2.00 (1.11)	2.04 (1.12)
Having sex with an adult female who hasn’t explicitly said no	2.69 (1.04)	2.07 (1.09)	1.95 (0.98)	2.44 (1.11)	2.06 (1.11)	2.15 (1.10)
Having sex with an adult female who is asleep	1.41 (0.89)	1.26 (0.56)	1.17 (0.50)	1.26 (0.74)	1.28 (0.64)	1.33 (0.88)
Overall composite score	1.78 (0.72)	1.53 (0.48)	1.40 (0.34)	1.63 (0.55)	1.51 (0.58)	1.54 (0.62)

Response options ranged from 1 (*Very unlikely*) and 5 (*Very likely*)

**Table 2:** Wilcoxon signed-rank tests for changes in outcome scores over time

Measure	Median difference (*r*)		
	Pre-test to post-test	Post-test to follow-up	Pre-test to follow-up
SPLS			
TG	− 0.29 (0.34)***	− 0.08 (0.27)***	− 0.28 (0.44)***
WCG	− 0.16 (0.22)***	0.02	− 0.13 (0.23)***
HTW			
TG	− 2.00 (0.31)***	− 1.09 (0.28)***	− 2.92 (0.44)***
WCG	− 1.00 (0.17)**	− 0.36	− 1.00 (0.25)***
IRMA-R			
TG	− 3.50 (0.42)***	− 1.15 (0.25)***	− 5.00 (0.50)***
WCG	− 0.88	− 1.33 (0.22)***	− 1.00 (0.24)***
SFQ-R-SV			
TG	− 1.00 (0.18)**	− 0.84 (0.20)**	− 1.06 (0.22)***
WCG	0.00	− 0.85 (0.20)**	0.00

SPLS = Self-Perceived Likelihood Scale; TG = treatment group; WCG = waitlist control group; HTW = Hostility Towards Women Scale; IRMA-R = Illinois Rape Myth Acceptance Scale—Revised; SFQ-R-SV = Sexual Fantasy Questionnaire Revised—Short Version

^**^p < .01 ***p < .001

#### Group × Time Interaction

To establish whether there was a difference in SPLS scores between groups over each of the three testing points, a two-way mixed ANCOVA was run with participants’ age and current level of university study specified as covariates.^6^ Mauchly’s test indicated that the assumption of sphericity was violated for the two-way interaction, *χ*^2^(2) = 90.76, *p* < 0.001; subsequently, a Greenhouse–Geisser correction was applied.

While there were no significant between or within-subjects effects, results showed a significant interaction between group allocation and time on SPLS scores, *F*(1.53, 383.01) = 11.94, *p* < 0.001, *ε* = 0.77, partial *η*^2^ = 0.05 (a small effect size). This indicates that variations in participants’ SPLS scores over time were determined by the group they were allocated to. Shifts were probed through a series of pairwise comparisons, which showed that TG participants displayed a moderate significant decline in their SPLS scores between pre-test and post-test, and also between post-test and follow-up. This suggests that TG participants’ proclivity towards sexual aggression was positively impacted by their participation in the intervention and that these treatment shifts continued for several months after the intervention ended. Pairwise comparisons also showed that WCG exhibited a significant decline in their SPLS scores between pre-test and post-test (albeit to a lesser degree than TG participants); however, unlike their counterparts, this trend did not continue beyond post-test.

#### Clinically Reliable Change

As shown in Table 3, the majority of TG and WCG participants were classified as “unchanged” at post-test and follow-up based on their SPLS scores, yet the proportion of “unchanged” WCG participants was significantly greater at follow-up compared to the TG. Although the proportion of participants classified as “recovered” or “reliably improved” was smaller, significantly more TG participants were “recovered” at follow-up and “reliably improved” at both post-test and follow-up. The “unreliably recovered” and “deteriorated” groups did not significantly differ between TG and WCG at both time points.

**Table 3:** Clinically reliable change for the Self-Perceived Likelihood Scale

Status	Treatment Group (*n* = 127)		Waitlist Control Group (*n* = 127)		Group classification at post-test		Group classification at follow-up	
	Pre-test to post-test *n* (%)	Pre-test to follow-up *n* (%)	Pre-test to post-test *n* (%)	Pre-test to follow-up *n* (%)	X^2^	*V*	X^2^	*V*
Recovered	3 (2.4%)	4 (3.1%)	4 (3.1%)	0 (0.0%)	0.15	.02	4.06*	.13*
Reliably Improved	12 (9.4%)	18 (14.2%)	3 (2.4%)	4 (3.1%)	5.74*	.15*	9.75**	.20**
Unreliably Recovered	22 (17.3%)	15 (11.8%)	24 (18.9%)	15 (11.8%)	0.12	.02	0.00	.00
Unchanged	83 (65.4%)	86 (67.7%)	93 (73.2%)	104 (81.9%)	1.85	.09	6.77**	.16**
Deteriorated	7 (5.5%)	4 (3.1%)	3 (2.4%)	4 (3.1%)	1.67	.08	0.00	.00

Figures may not add up to 100% due to rounding. *V* = Cramer’s V

^*^ p < .05 ** p < .01

### Secondary Intervention Outcomes

Table 4 displays the mean scores of TG and WCG participants on secondary outcome measures across testing points. Wilcoxon signed-rank tests are again shown in Table 2. Secondary outcome RCI score categories are shown in Supplemental Table 2–4.

**Table 4:** Mean scores across secondary outcome measures (SD)

Time	HTW		IRMA-R		SFQ-R-SV	
	TG	WCG	TG	WCG	TG	WCG
Pre-test	29.53 (11.03)	27.53 (9.68)	40.37 (13.58)	36.65 (10.99)	20.19 (14.92)	15.72 (11.32)
Post-test	27.77 (10.47)	26.31 (9.31)	36.59 (12.18)	36.51 (11.97)	18.79 (13.00)	16.47 (9.60)
Follow-up	26.49 (9.61)	25.92 (8.68)	35.42 (12.29)	34.88 (10.70)	17.72 (12.09)	15.66 (10.35)

HTW = Hostility Towards Women Scale; IRMA-R = Illinois Rape Myth Acceptance Scale—Revised; SFQ-R-SV = Sexual Fantasy Questionnaire Revised—Short Version; TG = treatment group; WCG = waitlist control group

#### Group × Time Interaction

Three separate two-way mixed ANCOVAs were run to establish whether there was a difference in mean HTW, IRMA-R, and SFQ-R-SV scores between groups over each of the three testing points. Participants’ age and current level of university study were again entered as covariates. The assumption of sphericity was violated for both the IRMA-R, *χ*^2^(2) = 38.35, *p* < 0.001, and the SFQ-R-SV, χ^2^(2) = 80.47, *p* < 0.001; therefore, significance levels for these models were interpreted with a Greenhouse–Geisser correction applied.

Results showed a significant interaction effect of group allocation and time across all three secondary outcome variables: *F*(2, 500) = 3.07, *p* = 0.047, partial *η*^2^ = 0.01 for the HTW, *F*(1.75, 437.54) = 13.99, *p* < 0.001, *ε* = 0.88, partial *η*^2^ = 0.05 for the IRMA-R, and *F*(1.57, 391.80) = 4.38, *p* = 0.02, *ε* = 0.78, partial *η*^2^ = 0.02 for the SFQ-R-SV. This indicates that variations over time in participants’ scores on these measures were determined by their group allocation. Shifts were probed through a series of pairwise comparisons, which showed that TG participants displayed small to large significant declines in their scores on all three measures between pre-test and follow-up, as well as small to moderate significant declines between both pre-test and post-test, and post-test and follow-up. While WCG participants also displayed a small reduction in their HTW and IRMA-R scores between pre-test and follow-up, only select pairwise comparisons were significant between pre-test and post-test, and post-test and follow-up. On the SFQ-R-SV, WCG participants only exhibited a small significant decline in their scores between post-test and follow-up.

Beyond interaction effects, there were no simple main effects of group across any of the three measures, nor a simple main effect of time for either the HTW or SFQ-R-SV. However, there was a significant effect of time on IRMA-R scores for the TG, *F*(1.56, 193.61) = 4.71, *p* = 0.02, *ε* = 0.78, partial *η*^2^ = 0.04 such that IRMA-R scores decreased over time. Effect sizes were small across all tests.

#### Clinically Reliable Change

RCI scores suggested that the majority of both TG and WCG participants did not exhibit a reliable change in secondary outcome measures at post-test and follow-up, with only a few differences observed between groups. Notably, a greater number of TG participants demonstrated reliable improvement in IRMA-R scores at post-test and in SFQ-R-SV scores at follow-up.

### Factors Predicting Intention to Complete the Intervention

A standard multiple regression was run to assess whether TG participants’ responses across the attitudes, subjective normative beliefs, and perceived behavioral control subscales of the TPBQ could predict their self-reported intention to complete the intervention (see Table 5). The resulting model was significant, *F*(3, 98) = 22.42, *p* < 0.001, *f *^2^ = 0.69. *R*^2^ for the overall model was 40.7% and adjusted *R*^2^ was 38.9%, a large effect size according to Cohen (1988). Of the variables that entered the model, only attitudes and perceived behavioral control scores made a significant contribution (*p* < 0.001 and *p* = 0.01, respectively). All participants who responded to the TPBQ completed the intervention.

**Table 5:** Factors predicting intention to complete the intervention among treatment group participants (n = 102)

Subscale	Β	*SE* Β	β	*sr*^2^	95% CI for Β	
					*LL*	*UL*
Attitude	0.40	0.08***	0.56	0.15	0.24	0.55
Perceived behavioral control	0.18	0.07**	0.22	0.04	0.04	0.32
Subjective normative beliefs	− 0.03	0.07	− 0.04	0.00	− 0.16	0.11
Constant	3.07	0.40	–	–	2.27	3.87

CI = confidence interval; B = unstandardized coefficient; *SE* B = standard error of the coefficient; β = standardized coefficient; *sr*^2^ = squared semi-partial correlation coefficient; *LL* = lower limit; *UL* = upper limit

^**^p < .01 ***p < .001

### Perceptions of the Intervention

Supplemental Table 5 provides an overview of responses to our user feedback measure. Though some participants found The Pathways program to be “common sense” and “repetitive,” the majority responded positively to it. Several participants said they were more confident engaging in healthy sexual activity having completed the program. Many also supported making the program—or a similar intervention—mandatory for students at their university. Helpful suggestions to improve the program included embedding additional examples of harmful sexual activity across exercises and including more challenging quizzes to reinforce module content.

## General Discussion

Contemporary research examining sexual harm prevention strategies at UK universities has shown that, despite recent scholarly advances, few interventions have been developed based on academic understanding of sexual perpetration by UK male students. Of the evidence-based interventions that do exist, most adopt a community-based approach to sexual harm prevention (e.g., bystander programs) and are developed using US data. Our paper contributes to the evolving research landscape by providing preliminary evidence for the feasibility and efficacy of a novel online self-help intervention for male sexual aggression that is grounded in academic understanding of university-based sexual aggression in the UK. Below we discuss our findings with reference to recent work in the field.

### Primary Outcome Analyses

Our findings showed that UK male students report varying levels of proclivity to engage in harmful sexual behaviors. To illustrate, participants typically rejected SPLS items regarding explicit forms of sexual aggression (e.g., rape) in favour of items that reflected lower-level sexually aggressive behaviors. These patterns mirror those reported by Wong et al. (2020) and support recent contentions that many male UK students are uncertain of the key hallmarks of valid sexual consent (e.g., Hills et al., 2020; Wignall et al., 2020).

Consistent with our hypothesis, mixed model testing showed that participating in The Pathways program led to reductions in participants’ self-reported likelihood to engage in sexual aggression over time. Specifically, pairwise comparisons highlighted moderate significant declines in SPLS scores at both post-test and follow-up for TG participants relative to notably smaller declines for WCG participants. These findings suggest promise for both the short and longer-term capability of our intervention to influence UK male students’ harmful sexual proclivities.

Based on CSC scores, the majority of TG and WCG participants were “unchanged,” meaning they did not demonstrate a significant change in SPLS scores. Yet, a significantly greater proportion of TG participants were classified as “recovered” at follow-up and a greater proportion were “reliably improved” at post-test and follow-up compared to the WCG. Although shifts in SPLS scores were smaller than anticipated, the intervention appears to have had a moderate effect on TG participants’ harmful sexual proclivities.

It is unclear why students in the WCG exhibited a reduction in their SPLS scores (albeit to a smaller degree), despite not having received our intervention. There are two likely explanations. First, that taking part in the pre-test survey encouraged participants to reflect on their sexual proclivities, which impacted their later responding. Second, that sexual proclivity naturally decreases as a student progresses through university. Further research is needed to explore these possibilities and determine the degree to which The Pathways program triggers, or perhaps accelerates, these shifts.

Our primary outcome analyses mirror those reported in evaluations of online sexual harm prevention programs for US university males (e.g., Salazar et al., 2014; Thompson et al., 2021). They also support Wong et al.’s (2020) findings, which illustrated greater positive shifts in SPLS scores among US male students who participated in a brief online self-persuasion intervention for sexual aggression versus those who did not. This evidence suggests that sexual proclivity is a malleable psychological trait that can be influenced through targeted intervention.

Though they did not comprise the majority, it is worth noting that four participants in the TG displayed an increase in their SPLS scores at follow-up. One possible explanation for this is offered by Malamuth et al. (2018), who show that “high-risk” university males (i.e., those most likely to engage in harmful sexual behaviors) often exhibit hostile reactance when they participate in sexual harm prevention programs. The authors reason that these students assume entitlement to have sex with women and, therefore, when presented with contrary evidence, display anger and hostility, compounding their likelihood of offending. Other UK university-based researchers have reported similar boomerang reactance effects (e.g., Fenton & Mott, 2018), suggesting this is a pervasive issue across sexual aggression research.

### Secondary Outcome Analyses

Beyond sexual proclivity, we also hypothesized that taking part in The Pathways program would lead to reductions in participants’ hostility towards women, RMA, and problematic sexual fantasies (see Hales & Gannon, 2022). Mixed model analyses confirmed our predictions, showing that the intervention positively impacted participants’ HTW, IRMA-R, and SFQ-R-SV scores over time. Specifically, pairwise comparisons showed that TG participants displayed small to large significant declines in their HTW, IRMA-R, and SFQ-R-SV scores at both post-test and follow-up testing. Contrary to predictions, follow-up tests showed that WCG participants also displayed significant reductions in their levels of hostility towards women and RMA at follow-up, though these were smaller shifts than those reported for TG participants. Based on RCI scores, the majority of TG and WCG participants did not display a reliable change in secondary outcome measures scores from pre-test to post-test or follow-up (i.e., they remained “unchanged”). However, a greater number of TG participants displayed a reliable improvement in SFQ-R-SV scores at follow-up compared to WCG participants. Significantly more TG participants also demonstrated a reliable improvement in IRMA-R scores at post-test, although this was not maintained at follow-up. We refer readers to the previous section for possible explanations for this.

Recent evaluations of other sexual harm prevention programs in the UK have reported positive post-intervention shifts in university students’ self-reported levels of RMA (e.g., Fenton & Mott, 2018; Roberts & Marsh, 2020; Thomson et al., 2020), suggesting that this is a trait that can be tackled effectively through targeted programing. To the best of our knowledge, there have been no interventions in the UK that have tried to challenge students’ hostile views towards women; however, US harm prevention studies have demonstrated promising treatment effects in this domain (e.g., Salazar et al., 2014). Likewise, harm prevention programs evaluated in the US or UK do not appear to have targeted university students’ problematic sexual fantasies. In this regard, the findings from our study contribute to a notable evidence gap.

### Program Completion

Similar to general sexual offence treatment evaluations (see Carl & Lösel, 2021; Olver & Wong, 2011), high levels of participant drop-out are common in longitudinal university-based sexual harm prevention studies (e.g., Salazar et al., 2014; Wong et al., 2020). Therefore, alongside our primary and secondary analyses, we also assessed the degree to which TG participants’ attitudes, subjective normative beliefs, and perceived behavioral control influenced their intention to complete The Pathways program. Using multiple regression analyses, we found that participants who possessed more positive attitudes towards intervention completion, as well as greater perceived self-control over their behaviors, reported a stronger intention to fully participate in the program. These findings suggest that promoting students’ self-efficacy—a key component of perceived behavioral control (Ajzen, 1991)—would likely empower them to in sexual harm prevention work, thus increasing their engagement in healthy sexual behaviors. Boosting self-efficacy could be achieved by discussing with students the benefits of program participation and positively reinforcing their willingness to contribute to campus safety (see Wojtowicz et al., 2013).

### Implications for Sexual Harm Prevention Work on UK Campuses

Presently, there is limited empirical evidence on effective sexual harm prevention strategies at UK universities. Alongside known issues with resource acquisition and institutional resistance (see Chantler et al., 2019; UUK, 2018), this lack of data has undoubtedly stalled the development of evidence-based interventions to tackle GBV on campuses nationwide. Camp et al. (2018), in their assessment of UK students’ views about sexual harm prevention, questioned the viability of short, one-time educational programs at tackling university-based sexual aggression and argued for more comprehensive prevention strategies. Similar concerns have been raised by academics in the US (e.g., DeGue et al., 2014; Vladutiu et al., 2011). Our findings challenge this notion by showing that participants who took part in The Pathways program demonstrated larger and longer-term attitudinal shifts relative to participants who had not taken part. Likewise, the fact that the intervention reduced participants’ post treatment levels of hostility towards women and RMA—established “hostile masculine” traits and likely indicators of “lad culture” (see Phipps & Young, 2013)—suggests that our intervention could be effective at combatting the risk factors for sexual aggression pertinent to male university students in the UK. However, given that WCG participants also displayed shifts in their attitudes over time, we caution readers that more robust evaluations of our intervention are necessary before we can endorse its expansion or rollout across broader male student cohorts.

### Limitations and Future Directions

In this paper, we present initial evidence regarding the feasibility and efficacy of The Pathways program at reducing university-based male sexual aggression in the UK. In doing so, we advance the field by offering preliminary empirical evidence in support of psychoeducation-based online self-help programs as a viable means of tackling university-based sexual aggression in the UK. However, despite this positive contribution, we are mindful that our study possesses some limitations that we urge readers to consider when interpreting our findings. We briefly outline these below.

First, participants self-selected to take part in our study. As such, there is a chance that we did not attract male students who possessed high levels of proclivity towards sexual aggression, who may have purposively avoided our research under fear of negative appraisal or punishment. Though we tried to mitigate against self-selection bias by fully anonymising our survey, this is a well-known issue that afflicts sexual harm prevention work (see Camp et al., 2018; Fenton & Mott, 2018). Mandating programing may provide a means to capture elusive students.

Second, based on the limited empirical evidence relating to UK male students’ harmful sexual behaviors, The Pathways program focusses only on psychological indicators of sexual perpetration among male students. However, we acknowledge that students operate as part of a multi-layered environment that includes influences from peers, their university, and wider society (see Bonar et al., 2022; Hales, 2022; McMahon et al., 2019; Spencer et al., 2023). To this end, we support the proposition of US academics (e.g., Bonar et al., 2022; Brennan et al., 2019; Vladutiu et al., 2011) and UUK (2016, 2018) that higher education providers need to adopt a multi-pronged approach to sexual harm prevention that includes a variety of evidence-based strategies to disrupt sexual perpetration by students. While there are currently no recommendations as to what constitutes effective prevention planning in the UK, recent research supports the use of bystander intervention training (e.g., Fenton & Mott, 2018; Roberts & Marsh, 2020), consent education (National Union of Students, 2015), and social norms alcohol initiatives (e.g., Bewick et al., 2008). Campus-wide marketing and media campaigns have also received positive academic evaluation (see Camp et al., 2018; Thomson et al., 2020). As noted in this paper, any initiatives need to be longitudinally evaluated to ensure that they deliver desirable outcomes. It would also be beneficial to examine outcomes against UK male students’ actual offending behaviors to examine whether as strong a link exists between their self-reported proclivity towards sexual aggression and their engagement in such acts as in US university cohorts.

Third, the high drop-out rate in our study meant that we were unable to capture our full sample of participants at post-test or follow-up (26.4% and 50.8% drop-out, respectively). Unfortunately, this is a common issue for intervention evaluation work in this space (e.g., Salazar et al., 2014; Wong et al., 2020). While high rates of drop-out can be indicative of systematic bias, the TG and WCG in our study did not differ on their levels of attrition. Additionally, our analyses used an intention-to-treat design which minimises, as much as possible, bias of this nature.

Fourth, US evidence suggests that university males who perpetrate sexual harm are a heterogenous group (e.g., Brennan et al., 2019; Swartout et al., 2015). Research is being conducted by the authors to evaluate this claim with UK students. If similar patterns are found, this would suggest that our “one-size-fits-all intervention” may not be suitable for all university males who report a proclivity towards sexual perpetration. This may also explain why we did not find superior reliable change indicators of program effectiveness for TG versus WCG participants. To this end, future research should consider developing screening tools to help professionals decide which modules potential aggressors will benefit from participating in, based on the specific risk factors associated with their harmful sexual proclivities. In the longer-term, these screeners could be embedded into prevention programs to provide tailored treatment options for students.

Fifth, there was a preponderance in our study towards White British students who were studying at a university in England. While our sample reflected the wider UK male student body (see HESA, 2022), it would be helpful to evaluate the efficacy of our intervention across more marginalized groups (e.g., ethnic minority students). It would also be interesting to expand our research agenda by examining program effectiveness among non-heterosexual UK student groups, to assess whether more targeted or specialised harm prevention interventions are necessary for the LGBT+ community.

Lastly, we acknowledge that our study attracted a high proportion of mature student participants (the average participant age was 26 years), including several (over 47%) who had a partner or wife. These demographic constraints limit the generalisability of our findings, particularly to younger students who comprise the majority of the male student cohort in the UK (see HESA, 2022). Subsequently, it would be helpful if future researchers explored the effectiveness of The Pathways program among a broader group of younger, single (particularly first and second year) UK male students, given evidence suggesting that this demographic is at increased risk of perpetration (e.g., Teten Tharp et al., 2013).

### Conclusion

The findings presented in this paper suggest that psychoeducation-based online self-help interventions may help combat sexual harm perpetration on UK campuses, albeit to a lesser degree than anticipated. In particular, The Pathways program—a novel evidence-based intervention designed around contemporary academic understanding of UK university-based sexual harm perpetration—appears to engender moderate positive attitudinal shifts among UK male students. Follow-up research is needed to validate the findings reported in this paper and assess in greater depth the efficacy of online self-help programs as effective primary prevention strategies for university-based sexual aggression in the UK. In attempting to replicate our findings, we would encourage researchers to trial other research designs and analytic strategies—as two examples, the use of an active control group and a per-protocol analysis—to provide additional insight into the intervention evaluation methods available to UK researchers working in this arena. Until such a time that academic understanding in the UK has improved, we would encourage UK universities to explore the possibility of integrating psychoeducation-based programs as part of a broader armoury against GBV only.

### Supplementary Information

Below is the link to the electronic supplementary material.

Supplementary file1 (DOCX 39 KB)

## Data Availability

The data and materials used in this study are available on the Open Science Framework at https://osf.io/b79n3/. Here, readers will also find details of our pre-registration.

## Footnotes

1 In this paper, we define “problematic” sexual fantasies as those that are coercive, deviant, or atypical in nature (e.g., paraphilic interests).

2 Initially, our pre-registration for this study covered only pre/post-intervention evaluation. However, additional funding was later sourced that enabled us to run the follow-up arm to our study. At this point, an amended pre-registration was uploaded to OSF.io.

3 Nondescript SPLS items were changed so that participants knew that victims were adult females. Likewise, to reflect UK law, the item “Having sex with a someone who is not sober” was amended to “Having sex with an adult female who is incapacitated.”

4 The participant’s pre-test responses were retained; however, consistent with intention-to-treat principles, their post-test and follow-up responses were imputed as they were not able to take part in these studies, having scored less than 70% in the intervention.

5 We do not refer to clinical significance for the “deteriorated” group since this refers to movement in a functional or positive direction.

6 We also conducted mixed model analyses excluding age and current level of university study as covariates. This revealed similar headline findings.
