# Supplementary material for: Empirically Assessing the Effectiveness of the Pathways Programme: An Online Self-Help Intervention for Male Sexual Aggression at UK Universities
Source: Arch Sex Behav. 2024 Feb 5;53(4):1377–94. doi: 10.1007/s10508-024-02808-6 (PMC10954925; doi:10.1007/s10508-024-02808-6)
Supplement: Supplementary file 1 — Supplementary file1 (DOCX 39 KB) [file 10508_2024_2808_MOESM1_ESM.docx]

**Supplementary Materials**

**Supplemental Table 1**

*Demographic Comparisons between Treatment Group and Waitlist Control Group Participants*

| Variable | TG (*n* = 127) | WCG (*n* = 127) |
| --- | --- | --- |
|  | *n* (%) | *n* (%) |
| Age ^a^ | | |
| 20 and under | 30 (23.6) | 32 (25.2) |
| 21-30 | 70 (55.1) | 66 (52.0) |
| 31-40 | 20 (15.8) | 24 (18.9) |
| 41-50 | 5 (3.9) | 3 (2.4) |
| 51-60 | 1 (0.8) | 2 (1.6) |
| 61-70 | - | - |
| 71-80 | 1 (0.8) | - |
| Ethnicity | | |
| White - English / Welsh / Scottish / Northern Irish / British | 66 (52.0) | 72 (56.7) |
| White - Irish | - | 3 (2.4) |
| White - Gypsy or Irish Traveller | - | - |
| White - Any other background | 26 (20.5) | 12 (9.5) |
| Mixed / Multiple ethnic groups - White and Black Caribbean | 1 (0.8) | - |
| Mixed / Multiple ethnic groups - White and Black African | 1 (0.8) | - |
| Mixed / Multiple ethnic groups - White and Asian | 2 (1.6) | 3 (2.4) |
| Mixed / Multiple ethnic groups - Any other background | 1 (0.8) | 1 (0.8) |
| Asian / Asian British - Indian | 7 (5.5) | 9 (7.1) |
| Asian / Asian British - Pakistani | 2 (1.6) | 4 (3.2) |
| Asian / Asian British - Bangladeshi | 3 (2.4) | 3 (2.4) |
| Asian / Asian British - Chinese | 4 (3.2) | 2 (1.6) |
| Asian / Asian British - Any other background | 6 (4.7) | 7 (5.5) |
| Black / African / Caribbean / Black British - African | 5 (3.9) | 6 (4.7) |
| Black / African / Caribbean / Black British - Caribbean | - | 1 (0.8) |
| Black / African / Caribbean / Black British - Any other background | - | - |
| Arab | 2 (1.6) | 3 (2.4) |
| Other / Prefer to self-describe | 1 (0.8) | 1 (0.8) |
| Current level of university study | | |
| Foundation stage or equivalent | - | 5 (3.9) |
| Undergraduate or equivalent | 82 (64.6) | 80 (63.0) |
| Master’s or equivalent | 32 (25.2) | 29 (22.8) |
| PhD / Doctoral or equivalent | 13 (10.2) | 13 (10.2) |
| Other ^b^ | - | - |
| Relationship status | | |
| Single or Self-partnered | 64 (50.4) | 69 (54.3) |
| In a relationship or Common law partnership | 52 (40.9) | 41 (32.3) |
| Married | 10 (7.9) | 16 (12.6) |
| In a civil partnership | 1 (0.8) | - |
| Divorced | - | - |
| Separated | - | - |
| Widowed | - | 1 (0.8) |
| Other / Prefer to self-describe | - | - |
| University country |  |  |
| England | 96 (75.6) | 100 (78.7) |
| Scotland | 13 (10.2) | 9 (7.1) |
| Wales | 7 (5.5) | 8 (6.3) |
| Northern Ireland | - | 1 (0.8) |
| Open University | 11 (8.7) | 9 (7.1) |

*Note.* Figures may not add up to 100% due to rounding. TG = treatment group; WCG = waitlist control group.

^a^ For ease of reading, participants’ ages have been grouped. Age was analysed as a continuous measure in our analyses.

^b^ The one WCG participant who (incorrectly) responded “Other / Prefer to self-describe” to this item was categorised into a pre-existing group.

**Supplemental Table 2**

*Reliable Change for Hostility Towards Women scale*

|  | Treatment Group  (*n* = 127) | | Waitlist Control Group  (*n* = 127) | | Group proportions at post-test | | Group classification at follow-up | |
| --- | --- | --- | --- | --- | --- | --- | --- | --- |
| Status | Pre-test to post-test  *n* (%) | Pre-test to follow-up  *n* (%) | Pre-test to post-test  *n* (%) | Pre-test to follow-up  *n* (%) | ꭓ^2^ | *V* | ꭓ^2^ | *V* |
| Improved | 5 (3.9%) | 4 (3.1%) | 6 (4.7%) | 8 (6.3%) | .10 | .02 | 1.40 | .07 |
| Unchanged | 120 (94.5%) | 122 (96.1%) | 119 (93.7%) | 117 (91.2%) | .07 | .02 | 1.77 | .08 |
| Deteriorated | 2 (1.6%) | 1 (0.8%) | 2 (1.6%) | 2 (1.6%) | .00 | .00 | .34 | .04 |

*Note.* Figures may not add up to 100% due to rounding. *V* = Cramer’s V.

**Supplemental Table 3**

*Reliable Change for Illinois Rape Myth Acceptance scale - Revised*

|  | Treatment Group  (*n* = 127) | | Waitlist Control Group  (*n* = 127) | | Group classification at post-test | | Group classification at follow-up | |
| --- | --- | --- | --- | --- | --- | --- | --- | --- |
| Status | Pre-test to post-test  *n* (%) | Pre-test to follow-up  *n* (%) | Pre-test to post-test  *n* (%) | Pre-test to follow-up  *n* (%) | ꭓ^2^ | *V* | ꭓ^2^ | *V* |
| Improved | 14 (11.0%) | 13 (10.2%) | 5 (3.9%) | 14 (11.0%) | 4.61* | .14* | .04 | .01 |
| Unchanged | 110 (86.6%) | 112 (88.2%) | 116 (91.3%) | 108 (85.0%) | 1.45 | .08 | .54 | .05 |
| Deteriorated | 3 (2.4%) | 2 (1.6%) | 6 (4.7%) | 5 (3.9%) | 1.04 | .06 | 1.32 | .07 |

*Note. * p* < .05. Figures may not add up to 100% due to rounding. *V* = Cramer’s V.

**Supplemental Table 4**

*Reliable Change for Sexual Fantasies Scale Revised - Short Version*

|  | Treatment Group  (*n* = 127) | | Waitlist Control Group  (*n* = 127) | | Group classification at post-test | | Group classification at follow-up | |
| --- | --- | --- | --- | --- | --- | --- | --- | --- |
| Status | Pre-test to post-test  *n* (%) | Pre-test to follow-up  *n* (%) | Pre-test to post-test  *n* (%) | Pre-test to follow-up  *n* (%) | ꭓ^2^ | *V* | ꭓ^2^ | *V* |
| Improved | 7 (5.5%) | 11 (8.7%) | 7 (5.5%) | 3 (2.4%) | .00 | .00 | 4.84* | .14* |
| Unchanged | 114 (89.8%) | 110 (86.6%) | 109 (85.8%) | 118 (92.9%) | .92 | .06 | 2.74 | .10 |
| Deteriorated | 6 (4.7%) | 6 (4.7%) | 11 (8.7%) | 6 (4.7%) | 1.58 | .08 | .00 | .00 |

*Note*. ** p* < .05. Figures may not add up to 100% due to rounding. *V* = Cramer’s V.

**Supplemental Table 5**

*Treatment Group Participants’ Responses to the User Feedback Measure (n = 102)*

| Item | *M* (SD) | *Mdn* |
| --- | --- | --- |
| This intervention could help educate students about healthy sexual behaviours. | 6.28 (0.91) | 6.50 |
| I think this is an important intervention. | 6.20 (1.23) | 7.00 |
| This intervention is good for starting conversations about how to promote healthy sexual behaviours. | 6.20 (0.95) | 6.00 |
| I would want other students studying at my university to take part in this intervention. | 6.13 (1.23) | 7.00 |
| I feel more confident in how to engage in healthy sexual behaviours after completing this intervention. | 5.57 (1.61) | 6.00 |
| I believe doing this intervention could be beneficial to me. | 5.19 (1.71) | 6.00 |
| I would want my university to implement this intervention with its students. | 5.90 (1.35) | 6.00 |
| I think this intervention is important to take part in because it can help me prevent others from being hurt. | 5.65 (1.43) | 6.00 |
| This intervention made me think. | 5.92 (1.25) | 6.00 |
| I think taking part in this intervention is useful for developing healthy sexual relationships. | 6.04 (1.10) | 6.00 |
| I think that taking part in this intervention could help me to avoid being involved in a sexual assault. | 5.20 (1.76) | 6.00 |
| I thought this was a boring intervention. ^a^ | 2.72 (1.63) | 2.00 |
| I enjoyed taking part in this intervention. | 5.25 (1.41) | 6.00 |
| This intervention was interesting to do. | 5.76 (1.10) | 6.00 |
| This intervention was fun to do. | 4.75 (1.44) | 5.00 |

*Note.* Response options ranged from 1 (*Not at all true*) to 7 (*Very true*).

^a^ This item was reverse-coded. Presented here are un-recoded scores

**Supplemental Appendix A**

**The Modified Theory of Planned Behaviour Questionnaire**

***Preamble:*** The following survey is designed to help us to understand how participants interact with the intervention. Please make sure to read the questions and response options carefully. Select the response that best describes your opinion.

|  | I intend to complete this online intervention in the next 4 weeks.* | | | | | | |
| --- | --- | --- | --- | --- | --- | --- | --- |
|  | **1** | **2** | **3** | **4** | **5** | **6** | **7** |
|  | Extremely agree | Agree | Slightly agree | Neither agree nor disagree | Slightly disagree | Disagree | Extremely disagree |
|  | For me, completing this online intervention over the next 4-weeks is… | | | | | | |
|  | **1** | **2** | **3** | **4** | **5** | **6** | **7** |
|  | Extremely worthless | Worthless | Slightly worthless | Neither worthless nor valuable | Slightly valuable | valuable | Extremely valuable |
|  | I think that completing this online intervention over the next 4-weeks will improve my understanding of healthy sexual behaviours.* | | | | | | |
|  | **1** | **2** | **3** | **4** | **5** | **6** | **7** |
|  | Extremely likely | likely | Slightly likely | Neither likely nor unlikely | Slightly unlikely | Unlikely | Extremely unlikely |
|  | For me, improving my understanding of healthy sexual behaviours is…* | | | | | | |
|  | **1** | **2** | **3** | **4** | **5** | **6** | **7** |
|  | Extremely important | Important | Slightly important | Neither important nor unimportant | Slightly unimportant | Unimportant | Extremely unimportant |
|  | The important people in my life would want me to complete this online intervention.* | | | | | | |
|  | **1** | **2** | **3** | **4** | **5** | **6** | **7** |
|  | Extremely agree | Agree | Slightly agree | Neither agree nor disagree | Slightly disagree | Disagree | Extremely disagree |
|  | When it comes to addressing healthy sexual behaviours, how much are you guided by the opinion of important people in your life? | | | | | | |
|  | **1** | **2** | **3** | **4** | **5** | **6** | **7** |
|  | Not at all guided | Unguided | Slightly unguided | Neither guided nor unguided | Slightly guided | Guided | Extremely guided |
|  | For me, completing this online intervention in the next 4 weeks will be… | | | | | | |
|  | **1** | **2** | **3** | **4** | **5** | **6** | **7** |
|  | Completely impossible | Impossible | Slightly impossible | Neither possible nor impossible | Slightly possible | Possible | Completely possible |
|  | How much control do you believe you have over completing this online intervention over the next 4 weeks? | | | | | | |
|  | **1** | **2** | **3** | **4** | **5** | **6** | **7** |
|  | Absolutely no control | No control | Slightly no control | Neither control nor no control | Some control | Control | Complete control |
|  | Think of an external factor that would make it difficult for you to complete this online intervention in the next 4 weeks (e.g., university, work, busy schedule, etc.). In the next 4 weeks, I expect that this factor will be… | | | | | | |
|  | **1** | **2** | **3** | **4** | **5** | **6** | **7** |
|  | Extremely demanding | Demanding | Slightly demanding | Neither demanding nor undemanding | Slightly undemanding | Undemanding | Extremely undemanding |
|  | In the next 4 weeks, how do you expect the demands of this factor will affect your ability to complete this online intervention? | | | | | | |
|  | **1** | **2** | **3** | **4** | **5** | **6** | **7** |
|  | Extremely difficult | Difficult | Slightly difficult | Neither difficult nor easy | Slightly easy | Easy | Extremely easy |

* Responses were reverse-coded prior to analysis.

**Supplemental Appendix B**

**Structure and Content Overview of The Pathways Programme**

| **User Engagement Survey** (*i.e.,* *Theory of Planned Behaviour Questionnaire*) |
| --- |
| **Module 1:** What is University-based Sexual Aggression? |
| - Brief introduction to the intervention - The definition of “university-based sexual aggression” - The prevalence of sexual aggression on university campuses - The consequences of university-based sexual aggression - The causes of university-based sexual aggression - **Quiz:** Multiple choice quiz (MCQ) |
| **Module 2:** The Law on Sexual Aggression |
| - Brief introduction to the law on sexual aggression - UK legislation relevant to sexual aggression - How are sexually aggressive offences prosecuted? - How sexual offences progress through the UK criminal justice system - Sexually aggressive behaviours prosecutable under UK law - Actus rea and mens rea - **Quiz:** Scenario-based MCQ |
| **Module 3:** What is Consent and Why is it Important? |
| - What is sexual consent? - The hallmarks of valid sexual consent - Important considerations when seeking valid sexual consent - When is sexual consent not valid? - Sexual consent: myths versus realities - **Quiz:** Scenario-based MCQ |
| **Module 4:** Managing Problematic Sexual Fantasies |
| - What are sexual fantasies? - Types of sexual fantasies - What are the benefits of sexual fantasies? - The dangerous side of sexual fantasies - Are problematic sexual fantasies common? - Why are problematic sexual fantasies bad? - How to assess whether you experience problematic sexual fantasies - **Quiz:** Scenario-based MCQ - **Activity:** Masturbatory reconditioning (for those with problematic sexual fantasies) |
| **Module 5:** Promoting Positive Attitudes towards Women |
| - Men’s hostility towards women - Examples of hostile sexist beliefs - Why do some men have hostile sexist beliefs? - What are the effects of men’s hostility towards women? - Promoting positive attitudes towards women - **Quiz:** Scenario-based MCQ - **Activity:** Scenario-based reflection |
| **Module 6:** Dispelling Rape Myths |
| - What are rape myths? - How prevalent is rape myth acceptance? - Types of rape myth - Why do some people accept rape myths? - The dangerous side of rape myths - **Quiz:** Rape myth sorting exercise - **Activity:** Scenario-based reflection |
| **[Optional] Module 7:** Mindfulness Meditation |
| **User Feedback Measure** |
